# Levels of Main Bacterial Phyla in the Gastrointestinal Tract of Sheep Depending on Parity and Age

**DOI:** 10.3390/ani11082203

**Published:** 2021-07-25

**Authors:** Jakub Smoliński, Natalia Szeligowska, Paulina Cholewińska, Katarzyna Czyż, Marzena Janczak

**Affiliations:** Institute of Animal Breeding, Wroclaw University of Environmental and Life Sciences, ul. Chełmońskiego 38 c, 51-630 Wroclaw, Poland; 119635@student.upwr.edu.pl (N.S.); katarzyna.czyz@upwr.edu.pl (K.C.); marzena.janczak@upwr.edu.pl (M.J.)

**Keywords:** sheep, microbiome, digestive tract, age, pregnancy

## Abstract

**Simple Summary:**

The ruminant microbiome is considered a specific ecosystem found within the gastrointestinal tract. A balanced intestinal microbiota is important not only for maintaining gut homeostasis, but also for regulating immune function and has a direct impact on the gut–brain axis. Factors, such as pregnancy, age, or genetics, can influence the microbial composition of the digestive system. The results of the study suggest that the parity, as well as the age of the animals, may affect the level of microorganisms in the digestive system.

**Abstract:**

During pregnancy and parturition, the homeostasis of the body is disturbed, and the immune system is undermined, which is associated with hormonal changes within the body. Recently, it has also been suggested that physiological and hormonal changes associated with pregnancy may affect the composition of the gastrointestinal microbiome. Therefore, the aim of this study was to determine the composition of the microbiome in the third month of pregnancy in sheep in their first and second parity. Eighteen females in total were selected for the experiment, and they were divided into two groups: primiparous (aged 1 year) and multiparous ones (aged 2 years). The animals were fed the same fodder, and did not show any disease symptoms. Fecal samples were collected individually from each female (*n* = 20), and then bacterial DNA isolation and real-time PCR were performed for the main bacterial phyla (Firmicutes, Bacteroidetes, Proteobacteria, Actinobacteria) and families (Lactobacillaceae and Clostridia). The obtained results showed the differentiation in the microbiome between the primiparous and multiparous ewes with respect to the following groups: Bacteroidetes, Proteobacteria, and Actinobacteria—the level was higher in the case of the primiparas. These results suggest that the parity and age of the females may affect the gastrointestinal microbiome, but further studies are recommended.

## 1. Introduction

The ruminant microbiome is considered a specific ecosystem found within the gastrointestinal tract. The close relationship between the microbiome–digestive system–host influences the development and health status of the animal. In addition to the rumen, the large intestine is also considered to be one of the most developed parts of the gastrointestinal tract in terms of microbiology, where the level of bacteria increases in the caudal direction [1,2,3].

In ruminants, bacteria are the most numerous [4,5], followed by archaeons, fungi, and protozoa. Firmicutes, Bacteroidetes, Proteobacteria, and Fibrobacter phyla are most abundant. Tenericutes and Actinobacteria are found in small numbers, which is caused by the plant diet of ruminants [4,5,6]. The most abundant bacterial phyla are primarily associated with fodder rich in crude fiber and polysaccharides [3,7].

The study by Moore et al. [8] on cattle showed similar bacterial phyla in the gastrointestinal tract in both non-pregnant and pregnant individuals, i.e., Firmicutes, Bacteroidetes, and Proteobacteria, which accounted for about 40, 35, and 10% of the sequence, respectively. It was also shown that the microbiome of the external cervix surface was significantly different from the microbiome of the amniotic fluid, intercotyledonary placenta, and placentome tissues. Interestingly, many bacterial species associated with postpartum uterine disease (i.e., *Trueperella* spp., *Acinetobacter* spp., *Fusobacteria* spp., *Proteus* spp., *Prevotella* spp., and *Peptostreptococcus* spp.) were also present in the uteri of cows that were not pregnant [8].

On the other hand, the study by Karstrup et al. [9] showed the presence of bacteria in the uterus of cows during pregnancy, which confirms the thesis that the uterine environment is not sterile. Studies on cows have shown that sterility is not maintained in the uterus during pregnancy. *Fusobacterium necrophorum, Porphyromonas levii*, and *Trueperella pyogenes* were found in the endometrium, on the endometrial surface, and in the caruncular stroma, despite the fact that no inflammation or disease were observed in the individuals, and these are typical bacteria associated with inflammation in the body. In addition, studies have indicated that a pregnant cow can carry the pregnancy to term despite several potentially pathogenic bacteria [8]. According to previous studies, the physiological state of pregnancy affects not only the microbiome of the reproductive system, but also the microbiome of the gastrointestinal tract, due to significant changes in the female body, including hormonal changes, development of the fetus, as well as changes in the diet during this period [10,11].

The studies carried out so far do not fully explain the operation of the microbiome in the perinatal period, especially in ruminants. Therefore, the aim of this study was to determine the levels of the main bacterial phyla of the gastrointestinal tract in relation to the parity and age in sheep.

## 2. Materials and Methods

### 2.1. Animals

The animals included in the study were Polish Pogórze sheep owned by a private farm situated in Lower Silesia province (Poland). Polish Pogórze sheep is a prolific breed (prolificacy of about 130%) developed in the 1950s in Poland. Since 2015, these sheep have been included in the Genetic Resources Conservation Programme in Poland due to their excellent adaptation to the conditions of the foothill regions of Poland [12].

On the selected farm, the sheep are used traditionally (1 lambing per year in February-March) and are housed on deep litter with access to pasture. The diet of the animals selected for the study was mainly based on oats (variety 00) about 300 g/head/day, ad libitum access to hay, water, and licks. Oats and hay came from the farm where the animals were also kept (the batch on which they were kept was fed for a period of two months). The animals selected for the study included 8 multiparous ewes and 8 primiparous ones, which were in the 3rd month of pregnancy. The animals did not show any disease symptoms.

### 2.2. Sampling Collection

Fecal samples were collected 1.5 months before parturition, individually from each ewe. They were collected directly after defecation (up to 10 s). They were then placed into a sterile container (60 mL) and frozen at −5 °C for the time of transport (20 min), and then stored at −26 °C until analysis (7 days).

### 2.3. Bacterial DNA (Deoxyribonucleic Acid) Isolation

Isolation of bacterial DNA was performed after each sample homogenization (by mixing) using a Genomic Mini AX Stool kit (A&A Biotechnology, Gdańsk, Poland) modified by the addition of mutanolysin and lysozyme. The amount of fecal samples needed for analysis was 100 µg. The concentration of DNA after isolation was then verified using a NanoDrop 2000 Spectrophotometer (Thermo Scientific, Wilmington, NC, USA). The average DNA in the samples of feces was 40–50 μg/μL (in 50 μL).), and the level of contamination in the samples was 2.0–2.2 for parameter 260/230 (contamination related to, among others, reagents used for the isolation) and 1.8–2.0 (correct levels, according to the instrument manual) for parameter 260/280 (contamination by substances, such as enzymes, inhibitors).

### 2.4. Real-Time PCR Analysis

A Bio-Rad CFX Connect 96 Touch apparatus was used to perform real-time PCR (Polymerase Chain Reaction) analysis. A Bio-Rad SsoAdvanced™ Universal SYBR^®^ Green Supermix kit (Bio-Rad Laboratories, Inc., Hercules, CA, USA) at a volume of 10 μL was applied in 3 technical repetitions. An NTC test (no template control) was additionally performed for each gene. The strategy of RT–PCR analysis involved the amplification of genes specific for the examined phyla in the presence of the reference gene for all bacteria (Table 1).

In order to determine the performance of individual genes, a standard curve was drawn for the genes under the study. A sample dilution of 10^−3^ was selected for the analysis (from the 10^−1^ to 10^−7^ series). The analysis was performed in accordance with a 40-cycle protocol: polymerase activation and DNA denaturation 95 °C (3 min), denaturation 95 °C (15 s), annealing 60.5 °C (15 s), and extension and plate reading at 72 °C (40 s). The melting curves analysis for the samples was performed at temperatures ranging from 65 (5 s) to 95 °C (0.5 °C increments in 2 s) [19]. 

The data obtained were then processed using the CFX Maestro software v. 1.1 (Bio-Rad Laboratories, Inc., Hercules, CA, USA). The sample with a DNA level of 100 μg/μL and impurities at a level consistent with the standards was an arbitrary calibrator. The efficiency of individual primers was normal (in accordance with the standards established by Bio-Rad) and amounted to 89.4% for Firmicutes, 93.6% for Actinobacteria, 97.7% for Lactobacillaceae; Universal—94.4. CFX Maestro calculated the results from the number of the reference gene matrix and the differences at the DNA level (ΔΔCq) and phylum’s level, taking into account the amplification efficiency of individual genes. 

### 2.5. Statistical Analysis

The results were analyzed with the use of the Statistica software (v. 13.3, StatSoft Inc., Tulsa, OK, USA). The data distribution was checked with the Shapiro–Wilk test. The data were analyzed using the Mann–Whitney U test (*p* < 0.05).

## 3. Results

**Figure 1:**
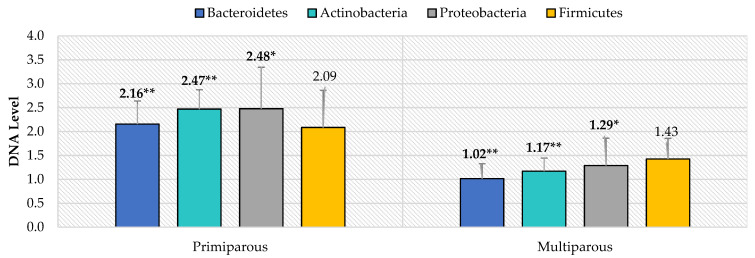
The level of selected bacterial phyla (RNE) in the gastrointestinal tract of ewes in relation to the parity based on feces (* *p* < 0.05; ** *p* < 0.01).

The real-time PCR analysis of the selected phyla revealed significant differences in terms of Bacteroidetes, Actinobacteria, and Proteobacteria levels (*p* < 0.05) depending on parity, i.e., between the primiparous and multiparous ewes (Figure 1). The primiparous ewes were characterized by significantly higher levels of most of the studied phyla, about two times compared to the multiparous ewes. However, there were no significant differences in the level of Firmicutes phylum depending on the parity.

**Figure 2:**
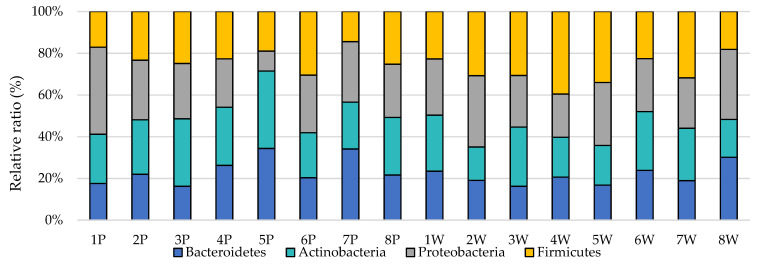
Relative ratio (%) of the chosen bacteria phyla in individual animals (P—primiparous, W—multiparous).

**Figure 3:**
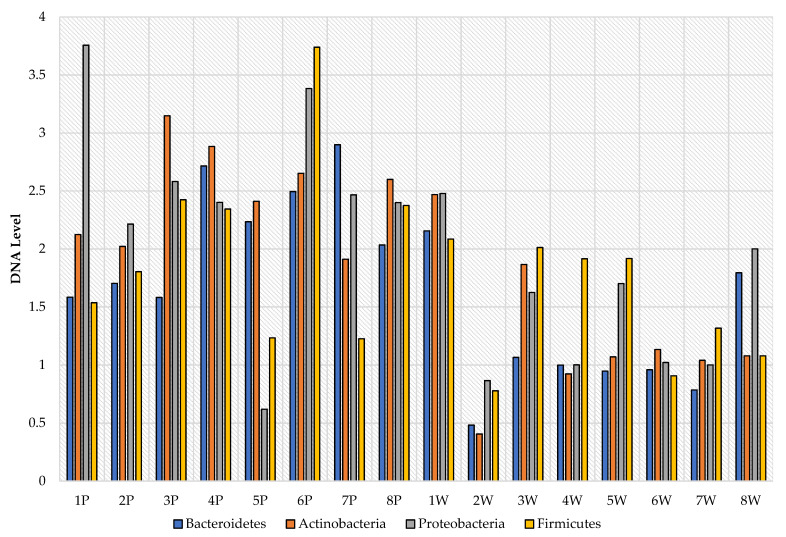
Individual levels of selected bacterial phyla (RNE) in the gastrointestinal tract of ewes based on feces (P—primiparous, W—multiparous).

**Figure 4:**
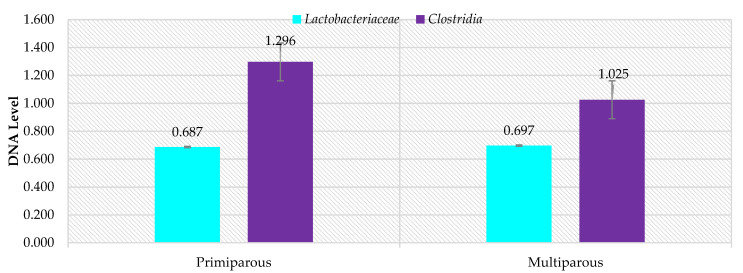
RNE levels of selected bacterial families in the gastrointestinal tract of ewes in relation to parity.

After also analyzing the levels of selected phyla from the individual point of view (host effect), it can be noticed that there were differences not only between the groups, but also individually (Figure 2 and Figure 3). Ewes in the first parity were characterized by quite a large variation in the levels of selected phyla, while in multiparous ewes, their levels were more equal. In addition, the RNE levels of the tested phyla were significantly higher in primiparous than in multiparous ewes, as shown in Figure 4, and this in turn was also confirmed by the earlier group analysis.

**Figure 5:**
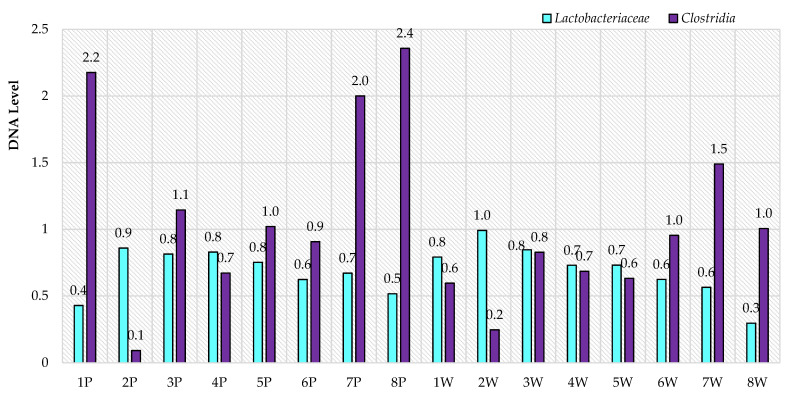
Individual levels of selected DNA bacterial families in the gastrointestinal tract of ewes based on feces (P—primiparous, W—multiparous).

In contrast, no differences were demonstrated for the *Lactobacillaceae* and *Clostridia* families (Firmicutes phylum) (*p* < 0.05). Levels were similar for both families (Figure 4) in terms of parity. However, there were differences between particular animals (Figure 5), where the most abrupt values were again found for primiparous ewes in terms of *Clostridia* family, compared to multiparous ewes. *Lactobacillaceae* family levels were similar in both primiparous and multiparous ewes.

## 4. Discussion

Ruminants are characterized by a high level of microbial system complexity. Factors, such as pregnancy, age, or genetics, can influence the microbial composition of the digestive system [10,20]. In the experiment conducted, primiparous ewes had significantly higher levels of the tested phyla than ewes in the second parity. Studies conducted in recent years suggest that the composition of the microbiome may also be influenced by hormones, particularly sex hormones [21,22]. In a study conducted by Markle et al. [23] on mice, it was demonstrated that there is a direct interaction between sex hormones and the gastrointestinal microbiome. The major female sex hormones include estrogens, where estrone, estradiol, and estriol are the most important ones. They are responsible for the maturation of the genital organs or the formation of secondary sexual characteristics. They also affect many metabolic pathways, including lipid metabolism, inhibition of gastrointestinal motility, and have an indirect effect on intestinal absorption, which is associated with the gastrointestinal microbiome [22,24,25]. Additionally, the study by Menon et al. [26] indicates that sex hormone levels may be important in gastrointestinal tract microbial variation. The study conducted on mice by Koern et al. [27] also showed that the microbiome in females during pregnancy changes significantly, as females during this time have reduced insulin sensitivity, which prepares them to build up energy stores for the rearing of offspring. In this study, it was found that there was a decrease in Firmicutes and Actinobacteria between the first and third trimesters of pregnancy in mice, with a concomitant increase in the Bacteroidetes level. However, the performed inoculation of the microbiome of pregnant mice in non-pregnant mice resulted in obesity.

The obtained differences in the levels of studied phyla between primiparous and multiparous ewes could also be related to the adaptation of the gastrointestinal microbiome and the changes occurring in it during pregnancy, which in turn may also be related to the age of females [20,28]. In the case of ruminants, changes/manipulations in the microbiome occur more easily in young animals compared to older ones, which may be related to the ability of the microbiome to adapt to both internal (physiological changes, including pregnancy) and external (under the influence of environmental factors) environments [29,30,31]. In the experiment, the ewes in the groups differed significantly in age: primiparous ewes were 12 months old while multiparous ewes were 2 years old, which means a year difference, so it can be supposed that adaptation of the microbiome to changes in hormone levels may be related to the age of the examined ewes; however, this requires further study. The microbiome in young individuals responds to dietary modification and physiological changes occurring in the host body. The results of studies conducted so far [4,5,6,7,8,9,20,29] suggest that manipulation of the microbiome in young animals is more effective and long term, which positively influences rumen development and, consequently, results in better gain and absorption of nutrients from feed. In contrast, adult individuals are not susceptible to long-term and effective microbiome manipulation. However, the improvement in the effectiveness of microbiome manipulation in both adult and juvenile individuals is not fully understood, suggesting further research on this topic Additionally, with age, the gastrointestinal microbiome of ruminants undergoes changes in its composition, where initial stabilization begins around 80 days of age; however, it is not associated with its full adaptation to environmental conditions as well as physiological changes [31,32,33,34]. Therefore, further studies are needed in terms of the influence of hormones and the parity on the gastrointestinal tract microbiome of ewes during pregnancy.

## 5. Conclusions

A balanced intestinal microbiota is important not only for maintaining gut homeostasis, but also for regulating immune function and has a direct impact on the gut–brain axis. The parity can affect the composition of the gastrointestinal microbiome and change its quantitative and qualitative composition. In the experiment conducted, the results suggest that the parity may influence the “response” of the microbiome to physiological phenomena occurring, which is also related to its adaptation to the prevailing conditions, both internal (host organism) and external ones (environment).

## Figures and Tables

**Table 1:** Primers used during RT–PCR analysis.

Name	Forward (5′-3′)	Reverse (5′-3′)
Universal Eubacterial Genes [13]	530F (5′-GTC CCA GCM GCN GCG G)	1100R (5′-GGG TTN CGN TCG TTG)
*Firmicutes* [14]	928F-Firm (5′-TGA AAC TYA AAG GAA TTG ACG)	1040FirmR (5′-ACC ATG CAC CAC CTG TC)
*Bacteroidetes* [14]	798cfbF (5′-CRA ACA GGA TTA GAT ACC CT)	cfb967R (5′-GGT AAG GGT TCC TCG CGT AT)
*Proteobacteria* [15]	27F (5′-GAGTTTGATCMTGGCTCAG-3′)	1529R(5′-CAKAAAGGAGGTGATCC-3′)
*Actinobacteria* [16]	Act1159R TCCGAGTTRACCCCGGC	Eub338F ACGGGCGGTGTGTACA
*Lactobacillaceae* [17]	lac1 forward (5′-AGC AGT AGG GAA TCT TCC A)	Lac2Seq (5′-ATTTCACCGCTACACATG)
*Clostridia* [18]	Clos58-fAAAGGAAGATTAATACCGCATAA	Clos780-r ATCTTGCGACCGTACTCCCC

## Data Availability

The data presented in this study are available on request from the corresponding author. The data are not publicly available due to privacy.
